# Applications of genotyping-by-sequencing (GBS) in maize genetics and breeding

**DOI:** 10.1038/s41598-020-73321-8

**Published:** 2020-10-01

**Authors:** Nan Wang, Yibing Yuan, Hui Wang, Diansi Yu, Yubo Liu, Ao Zhang, Manje Gowda, Sudha K. Nair, Zhuanfang Hao, Yanli Lu, Felix San Vicente, Boddupalli M. Prasanna, Xinhai Li, Xuecai Zhang

**Affiliations:** 1 Institute of Crop Sciences, Chinese Academy of Agricultural Sciences, Beijing, China; 2 International Maize and Wheat Improvement Center (CIMMYT), Apdo. Postal 6‑641, 06600 Mexico, DF Mexico; 3 Maize Research Institute, Sichuan Agricultural University, Wenjiang, Sichuan China; 4 CIMMYT-China Specialty Maize Research Center, Shanghai Academy of Agricultural Sciences, Shanghai, China; 5 Crop Breeding and Cultivation Research Institute, Shanghai Academy of Agricultural Sciences, Shanghai, China; 6 Agronomy College, Shenyang Agricultural University, Shenyang, Liaoning China; 7 International Maize and Wheat Improvement Center (CIMMYT), Village Market, P. O. Box 1041, Nairobi, 00621 Kenya; 8 CIMMYT-India, C/O ICRISAT, Patancheru, 502324 Andhra Pradesh India

**Keywords:** Plant breeding, Plant genetics

## Abstract

Genotyping-by-Sequencing (GBS) is a low-cost, high-throughput genotyping method that relies on restriction enzymes to reduce genome complexity. GBS is being widely used for various genetic and breeding applications. In the present study, 2240 individuals from eight maize populations, including two association populations (AM), backcross first generation (BC1), BC1F2, F2, double haploid (DH), intermated B73 × Mo17 (IBM), and a recombinant inbred line (RIL) population, were genotyped using GBS. A total of 955,120 of raw data for SNPs was obtained for each individual, with an average genotyping error of 0.70%. The rate of missing genotypic data for these SNPs was related to the level of multiplex sequencing: ~ 25% missing data for 96-plex and ~ 55% for 384-plex. Imputation can greatly reduce the rate of missing genotypes to 12.65% and 3.72% for AM populations and bi-parental populations, respectively, although it increases total genotyping error. For analysis of genetic diversity and linkage mapping, unimputed data with a low rate of genotyping error is beneficial, whereas, for association mapping, imputed data would result in higher marker density and would improve map resolution. Because imputation does not influence the prediction accuracy, both unimputed and imputed data can be used for genomic prediction. In summary, GBS is a versatile and efficient SNP discovery approach for homozygous materials and can be effectively applied for various purposes in maize genetics and breeding.

## Introduction

Genetic diversity analysis, linkage mapping, and association mapping, especially at the whole-genome level, form the foundation of modern molecular breeding^1^. Traditional molecular marker techniques can be used for genetic and breeding analyses to increase our understanding of complex quantitative traits, facilitate marker-assisted selection, and increase the efficiency. From restriction fragment length polymorphisms (RFLPs) to simple sequence repeats (SSRs) and single nucleotide polymorphisms (SNPs), the types of molecular markers predominantly used have evolved over the past several decades. SNPs are now widely used in genetic analysis and breeding. Large-scale genotyping at the whole-genome level is becoming increasingly important for understanding the genetic architecture of complex traits^2^. Sequencing technology improved greatly over the last four decades, with technical advances from Sanger sequencing to nanopore sequencing. Next-generation sequencing (NGS) technology has transformed modern biology with its high-throughput and low cost^3^. Multiplexing and sequencing-by-synthesis (SBS) have significantly improved the efficiency of sequencing^4^. NGS is comprised of three basic steps: (1) genomic DNA fragment library construction; (2) DNA amplification by polymerase chain reaction (PCR); and (3) sequencing^5^. Three major NGS platforms: 454 (Roche), Solexa (Illumina) and SOLID (ABI), have allowed wider application of genome sequencing. While each of these platforms has specific advantages and disadvantages, the choice of sequencing platform depends on the experimental purpose^6^.

The two strategies for NGS include whole-genome resequencing (WGR) and reduced-representation sequencing (RRS)^7^. The major difference between these two approaches is multiplex sequencing; and further, the cost of RRS per sample is much cheaper than that of WGR, which benefits from being largely unaffected by RRS biases^8^. Genotyping-by-sequencing (GBS) is one of most widely used RRS methods, where in the barcode system was improved to allow discovery genome-wide SNPs with a lower error rate and low cost^9^. At least 15 restriction enzymes now are available for GBS for use in sequencing the genomes even when the reference genome is not available^10^. The restriction enzyme *Ape* KI has been frequently used in GBS. A two-enzyme approach has been developed for GBS^11^. Sequence data software and pipelines have also been developed to improve the efficiency and versatility of GBS for SNP discovery and mapping^10,12,13^. For example, UNEAK (Universal Network Enabled Analysis Kit) is a network-based SNP calling pipeline for species without a reference genome, such as Switchgrass (*Panicum virgatum* L.)^14^. However, the inherently low genome coverage of GBS, which result in a high level of missing SNPs, has become a major bottleneck to its application. Therefore, data imputation pipelines such as FILLIN (Fast, Inbred Line Library ImputatioN) and FSFHap (full-sib families HapMap)^15^, as well as new bioinformatics methods like practical haplotype graph (PHG)^16^ have been developed to solve this problem.

The high-throughput SNPs detected by GBS are widely used for genetic diversity analysis^17–19^, genome-wide association studies (GWAS)^20–22^, QTL mapping^23–25^, and genomic prediction (GP)^26–28^ in many plant species. In the case of maize, GBS has been extensively applied for the sequencing of more than 17,000 maize materials (https://www.panzea.org/). Three generations of maize haplotype maps have constructed using these data^29^ and have been applied to studies of many aspects of maize genetics. For example, molecular characterization of 538 CMLs (CIMMYT Maize Lines) was undertaken using GBS, and three major subgroups and heterotic patterns in each group were identified^30^. Analysis of 8000 maize lines led to the identification of 220 candidate genes and 90 genomic regions related to flowering time, providing a good understanding of the genetic architecture of flowering time^31^. Genomic prediction (GP) for grain yield was performed at CIMMYT’s maize breeding program in Africa, genotyping 2022 breeding lines in Stage-1 yield trials by GBS. Compared to the pedigree-based method, GP has significant advantages for selecting for grain yield^32^. However, the main difficulty with the practical application of GBS is still the high rate of missing genotypes. For instance, only ~ 66,000 out of over 95,000 SNPs could be used in the GP study mentioned above after discarding markers with high rates of missing data^7,9^.

GBS has been applied to several studies on maize worldwide and has provided a great deal of information for researchers using GBS or similar RRS technologies^9,33^. In the present study, 2240 individuals from eight tropical maize populations developed at CIMMYT were analyzed using GBS with the following objectives: (i) to assess the efficiency of SNP discovery by GBS for different types of populations; (ii) to ascertain the utility of GBS data in genetic diversity analysis, GWAS, linkage mapping, and GP; and (iii) to determine the effects on genetic analysis of imputing genotype data.

## Results

### SNP-based analysis of eight tropical maize populations

In the present study, eight tropical maize populations developed at CIMMYT and consisting of 2240 individuals in total were analyzed using GBS after digesting genomic DNA with the restriction endonuclease *Ape* KI creating 96 or 384-plex libraries. These eight tropical maize populations could be classified in three ways: as a) an association mapping panel; b) segregating bi-parental populations (F2, BC1, BC1F2); and c) stabilized bi-parental populations (DH, IBM, RIL) (Table 1).

**Table 1:** Information about maize populations analyzed in the present study.

Pop	Type	Parent 1	Parent 2	Number of samples	Plex	Heterozygosity rate^a^ (%)	MAF^b^
Pop1	AM^c^	–	–	267	96	0.00	0 ~ 0.50
Pop2	AM	–	–	523	96	0.00	0 ~ 0.50
Pop3	BC1F2	DTPC9F104	CML491	174	96	25.00	0.25
Pop4	BC1	CKL09001	CML444	152	384	50.00	0.25
Pop5	F2	CLWN201	CML494	423	96	50.00	0.50
Pop6	DH	LPSC7F64	CML495	209	96	0.00	0.50
Pop7	RIL	B73	CML247	207	384	0.00	0.50
Pop8	IBM	B73	Mo17	285	384	0.00	0.50

^a^Expected heterozygosity rate of population.

^b^Expected minor allele frequency of population.

^c^Association panel.

### Genetic characteristic analysis using unimputed databases

For the association panel, the numbers of SNPs decreased from ~ 0.95 million to ~ 0.15 million after removal of SNPs with missing genotype rates > 50% and MAF < 0.05 (Table 2). The proportion of insertion/deletion variations decreased to less than 1% after filtering. The rates of missing SNP genotypes also decreased by more than 20% and MAFs increased by 0.14 after filtering for Pop1 and Pop2. The average heterozygosity rates of the two populations increased but were still less than 1% (Table 2).

**Table 2:** Information about unimputed SNPs detected in eight maize populations before and after data filtering.

Pop	Number of taxa		SNPs		Insertion/deletion (%)		Missing (%)^a^		Het (%)^b^		MAF^c^	
	Unfiltered	Filtered	Unfiltered	Filtered	Unfiltered	Filtered	Unfiltered	Filtered	Unfiltered	Filtered	Unfiltered	Filtered
Pop1	267	242	955,120	167,617	1.42	0.92	54.60	32.73	0.22	0.83	0.09	0.23
Pop2	523	513	955,120	115,311	1.46	0.78	60.83	33.48	0.11	0.56	0.09	0.24
Pop3	174	161	45,098	40,491	2.32	1.97	23.56	20.37	9.50	10.50	0.25	0.25
Pop4	152	152	41,307	13,662	1.88	1.22	57.44	31.85	5.46	12.68	0.27	0.27
Pop5	411	408	66,725	57,411	2.42	2.31	24.74	18.99	20.23	23.17	0.43	0.43
Pop6	207	177	65,814	48,985	2.73	1.99	21.03	16.36	0.86	0.87	0.35	0.43
Pop7	207	185	75,961	19,089	1.32	0.57	57.96	34.88	0.68	1.56	0.41	0.44
Pop8	285	216	73,013	36,468	1.26	0.88	49.55	36.13	0.70	1.00	0.40	0.42

^a^Percentage of missing SNP.

^b^Percentage of heterozygous SNP.

^c^Minor allele frequency.

For the bi-parental populations, the number of SNPs ranged from 41,307 to 75,961 before filtering (Table 2), much lower than for the association panels, which indicating the presence of greater genetic variation in the association panels than in the bi-parental populations. The number of SNPs changed little after filtering, except in Pop4, Pop7, and Pop8, which were sequenced using 384-plex libraries and exhibited higher missing SNP genotype rates. MAFs were maintained for all populations, except for Pop4 (Table 2).

### Genetic characteristic analysis using imputed databases

For the association panels, the numbers of SNPs decreased from ~ 0.95 to ~ 0.34 million after removal of SNPs with missing rates > 50% and MAF < 0.05 (Table 3). The proportion of insertion/deletion variants was reduced to about 0.50% after filtering. The rates of missing SNP genotypes greatly decreased substantially to less than 20% for the two association panels after imputation but changed little after filtering. However, the average MAF was 0.24, or 0.14 higher than in the unimputed data, indicating that most of the removed SNPs exhibited low MAF rather than high missing SNP genotype rate. The average heterozygosity rates of the two association panels increased after imputation but were still lower than 1% (Table 3).

**Table 3:** Imputed SNP information for eight populations before and after data filtering.

Pop	Number of taxa		SNPs		Insertion/deletion (%)		Missing (%)^a^		Het (%)^b^		MAF^c^	
	Unfiltered	Filtered	Unfiltered	Filtered	Unfiltered	Filtered	Unfiltered	Filtered	Unfiltered	Filtered	Unfiltered	Filtered
Pop1	242	242	955,120	341,312	0.82	0.62	15.20	12.75	0.94	2.30	0.09	0.23
Pop2	513	513	955,120	340,177	0.92	0.47	13.49	12.17	0.64	1.58	0.09	0.24
Pop3	161	161	93,760	76,437	1.48	0.74	8.44	2.86	19.40	21.26	0.24	0.24
Pop4	152	152	92,752	90,655	0.95	0.66	9.02	7.91	34.18	34.41	0.24	0.25
Pop5	408	408	91,564	90,637	1.88	1.74	4.07	3.66	43.10	43.67	0.45	0.45
Pop6	177	177	91,208	74,487	1.38	1.73	2.89	1.94	0.78	0.91	0.35	0.43
Pop7	185	185	121,935	121,013	1.78	0.71	2.45	2.11	5.36	5.39	0.45	0.45
Pop8	216	216	111,568	110,422	1.02	0.76	4.17	3.84	2.78	2.80	0.42	0.42

^a^Percentage of missing SNP.

^b^Percentage of heterozygous SNP.

^c^Minor allele frequency.

For the bi-parental populations, the number of SNPs ranged from 91,208 to 121,935 before filtering the data (Table 3), almost double to the number of SNPs in the unimputed data. The number of SNPs changed little after filtering, except for Pop3 and Pop6. For Pop3, the number of SNPs decreased due to the rate of missing SNP genotypes, which had fallen by ~ 66% after filtering the data. However, for Pop6, the number of SNPs decreased due to an increase in the MAF, which had risen by about 0.08 (Table 3). Moreover, the heterozygosity rates of the three segregating populations (Pop3, Pop4, and Pop5) were close to the theoretical value after imputation, while the heterozygosity rates for the other populations stayed fairly low (Tables 1 and 3), indicating that the imputation method was accurate and efficient across populations.

### Genotyping error

The genotyping errors for 955,210 unimputed and imputed SNPs were tested in all bi-parental populations. For the unimputed data, the average error rate for parents was 0.70%, which was much lower than for the F1 generations (Table 4). CML495 in Pop6 exhibited the highest error rate of 1.06%, while CML247 in Pop7 showed the lowest error rate of 0.51%. The error rates for heterozygous loci were 0.83 to 7.00 times greater than those for homozygous loci, which made using GBS for SNP calling of homozygous loci much more accurate than for heterozygous loci.

**Table 4:** Genotyping error rate of six bi-parental populations.

Pop	Line	All loci		Homozygous loci		Heterozygous loci	
		Unimputed (%)	Imputed (%)	Unimputed (%)	Imputed (%)	Unimputed (%)	Imputed (%)
Pop3	DTPC9F104	0.85	0.57	0.30	0.05	0.55	0.52
	CML491	0.87	0.31	0.25	0.12	0.62	0.19
	F1	8.21	10.42	3.38	0.68	4.83	9.74
Pop4	CKL09001	0.82	0.25	0.27	0.11	0.55	0.14
	CML444	0.67	0.18	0.18	0.08	0.49	0.10
Pop5	CLWN201	0.85	0.39	0.14	0.04	0.71	0.35
	CML494	0.61	0.33	0.09	0.07	0.52	0.26
	F1	7.97	5.74	2.43	0.38	5.54	5.36
Pop6	LPSC7F64	0.63	0.46	0.07	0.08	0.56	0.38
	CML495	1.06	0.74	0.17	0.09	0.89	0.65
Pop7	B73	0.56	0.38	0.16	0.19	0.40	0.19
	CML247	0.51	0.38	0.06	0.06	0.45	0.32
Pop8	B73	0.56	0.38	0.16	0.19	0.40	0.19
	Mo17	0.53	0.35	0.10	0.13	0.43	0.22
Average	Total	1.76	1.49	0.55	0.16	1.21	1.33
	Parents	0.70	0.45	0.16	0.11	0.54	0.33
	F1	8.09	8.08	2.91	0.53	5.19	7.55

The error rate for the imputed data was lower than for the unimputed data, with an average of 0.45% for parental lines due to a low rate of missing data after imputation. The line with the highest error rate was CML495, while the lowest error rate of 0.18% was recorded for CML444 (Table 4). The average error rate for heterozygous loci in parental lines was 0.33%, which was twofold that in the homozygous loci.

### Population structure of eight populations

**Figure 1:**
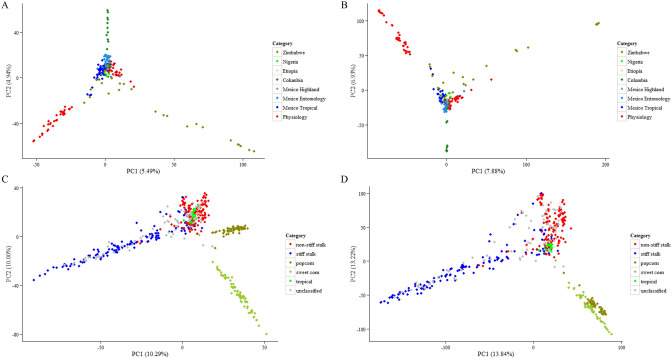
Principal component analysis of Pop1 and Pop2 using unimputed and imputed data. (**A**) Pop1 using unimputed data; (**B**) Pop1 using imputed data; (**C**) Pop2 using unimputed data; (**D**) Pop2 using imputed data.

Both unimputed and imputed data from eight populations were used to observe the impact of imputation on population structure analysis using PCA and multidimensional scaling (MDS). When using unimputed data, different subgroups could be separated by PCA in both association panels (Fig. 1A,C). For Pop1, clusters of lines from CIMMYT-Columbia, CIMMYT-Zimbabwe, and some CIMMYT-Physiology lines extended in three directions, while others were concentrated in the middle (Fig. 1A), which was consistent with the observations in a previous study^34^. For Pop2, different subgroups clustered along the PC1 axis, with popcorn and sweet corn on one side, and the non-stiff stalk lines on the other side. The stiff stalk and tropical lines could not be separated by the first two PCs (Fig. 1C), which was in congruent with Romay’s study^35^. When using imputed data, the two PCs explained more information but the distribution of the lines was basically the same for Pop1 and Pop2 (Fig. 1B,D).

**Figure 2:**
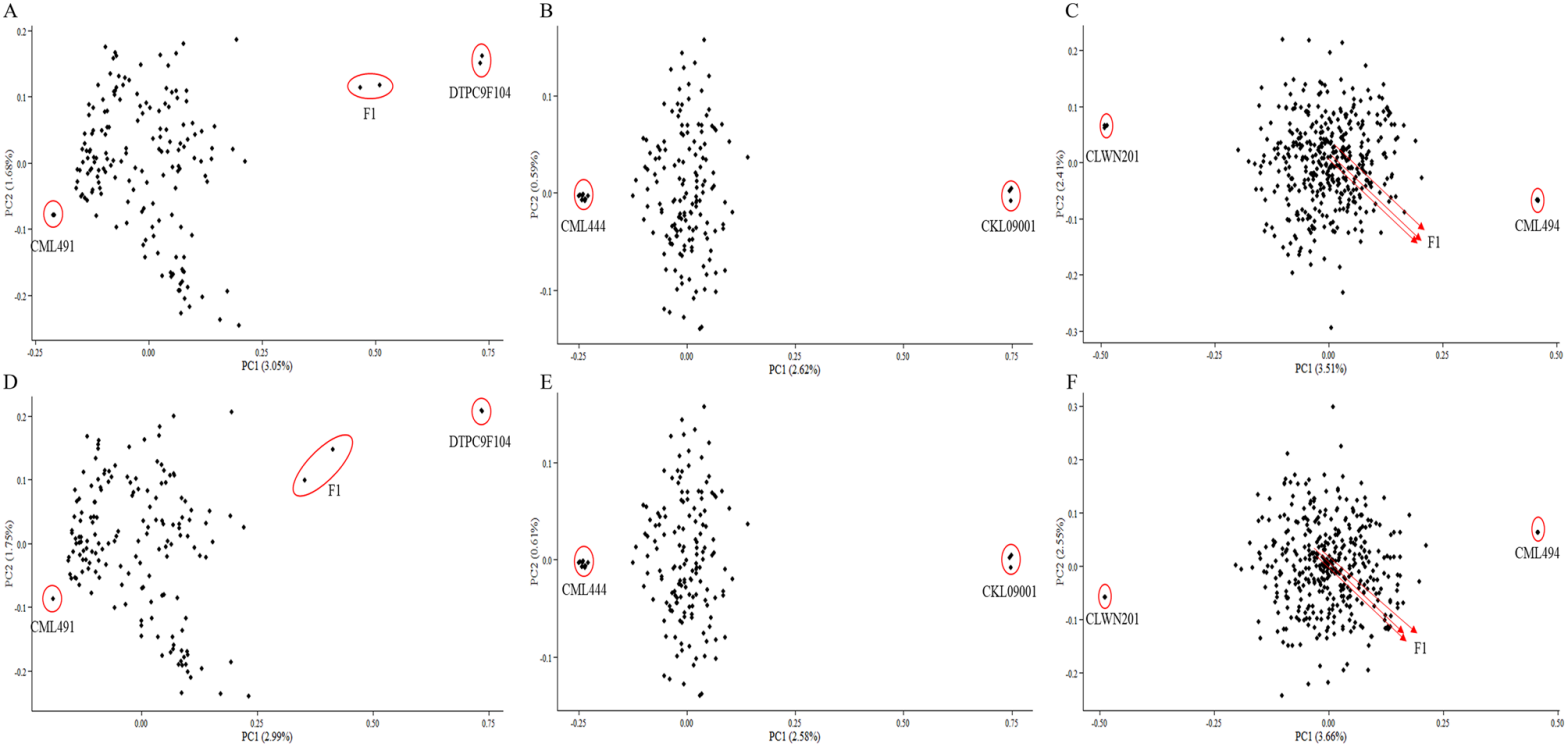
Multidimensional scanning for three bi-parental populations with high heterozygosity rate. (**A**–**C**) Pop3-5 using unimputed data; (**D**–**F**) Pop3-5 using imputed data.

For Pop3, Pop4, and Pop5, the MDS results showed clear relationships between the parental lines and progenies (Fig. 2). The filial generations were biased towards one parent in Pop3 (CML491) and Pop4 (CML444), as they had been backcrossed to the parent (Figs. 1B and 2A). While the progenies of Pop5, including the F1 hybrids, was balanced between the two parents (Fig. 2C). Moreover, the repeated parental lines overlapped, especially for CML491 (Fig. 2A). The results of these analyses were similar when using imputed data (Fig. 2D–F).

**Figure 3:**
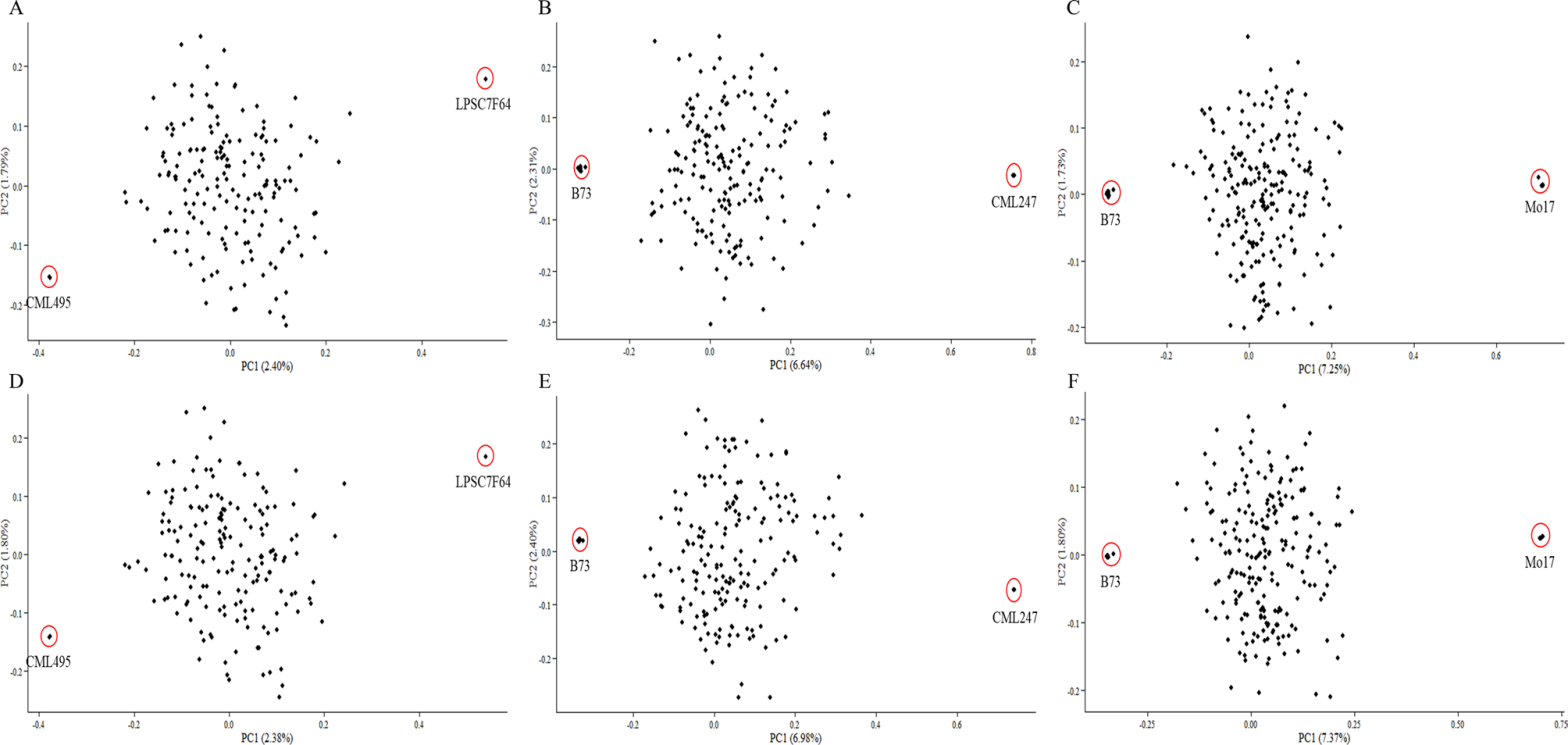
Multidimensional scanning for three bi-parental populations with low heterozygosity rate. (**A**–**C**) Pop6-8 using unimputed data; (**D**–**F**) Pop6-8 using imputed data.

Results of MDS were very similar for the three bi-parental populations with low heterozygosity rate (Pop6, Pop7, and Pop8) (Fig. 3). In addition, the parental lines were located closer when using imputed data (Fig. 3E,F). These results indicated that the population structure analysis was not influenced by the presence of unimputed or imputed data.

### GWAS of kernel color

**Figure 4:**
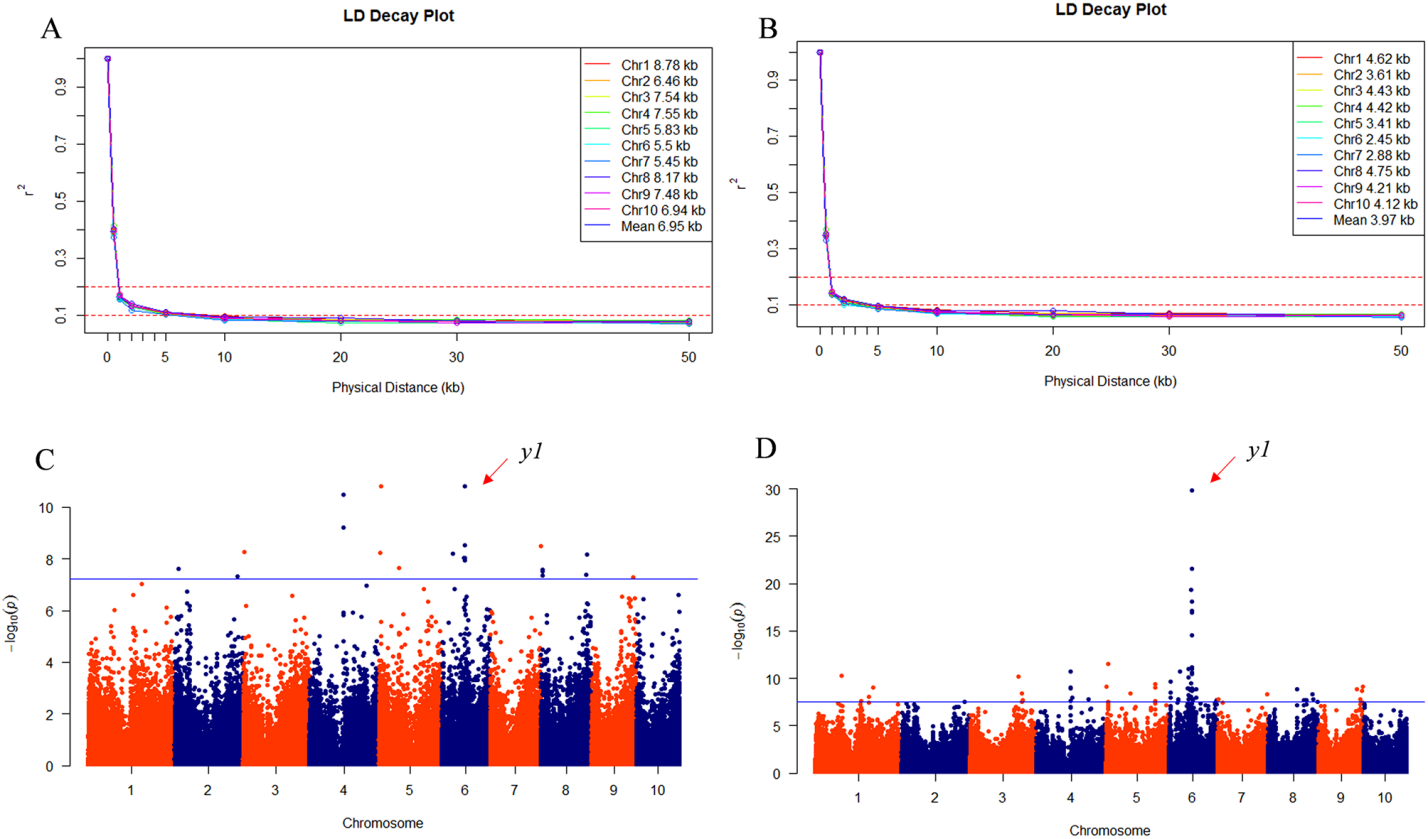
Decrease in linkage disequilibrium (LD) and GWAS for kernel color of Pop1 using filtered unimputed (**A**, **C**) and imputed (**B**, **D**) SNP data.

In the present study, kernel color in Pop1 was used to model mapping power and resolution in GWAS when using imputed or unimputed SNP data. Linkage disequilibrium (LD) decay occurred more rapidly by an average of 3.97 kb among all maize chromosomes (r^2^ = 0.1) when including imputed data, while the average LD unimputed was 6.95 kb using only unimputed data (Fig. 4A,B). These results indicate that more markers are needed to perform GWAS when using imputed SNP data. Considering the genome size of maize, about 520,000 markers were needed for effective GWAS when using imputed data, while 297,000 markers were needed when using only unimputed data, respectively.

Using only unimputed data, 24 SNPs associated with kernel color were detected that explained an average of 14% of the variance in this trait. Three SNPs with – log10(P) > 7.22 (0.01/n) were identified on chromosomes (Chrs.) 4, 5, and 6 (Fig. 4C). A set of 102 significant associated SNPs were identified at – log10(P) > 7.53 when using imputed data; half of these were located on Chr. 6, including the peak marker (Fig. 4D) SNP S6_82015505, which explained 30% of the variance in kernel color.

The *y1* gene has a major influence on the presence of β-carotene in maize endosperm^36^, an effect that could be clearly detected when using either imputed or unimputed data (Fig. 4C,D). The physical location for *y1* is from bp 82,017,148 to 82,021,007 on Chr. 6, according to the B73 AGPv2 coordinates. When only unimputed SNP genotype data were used in our analysis, the peak signal was detected within this region at 82,019,628 bp. When using imputed data, the most closely associated marker was SNP S6_82015505, approximately 1.5 kb away from the gene region, but within the average LD distance for Chr. 6 (Fig. 4B). However, the association signals identified using imputed data were much stronger than those identified using only unimputed data, suggesting that the imputed data in our study provided higher mapping resolution.

### Linkage mapping analysis using GBS data in a population with a relatively high genotyping error rate

**Figure 5:**
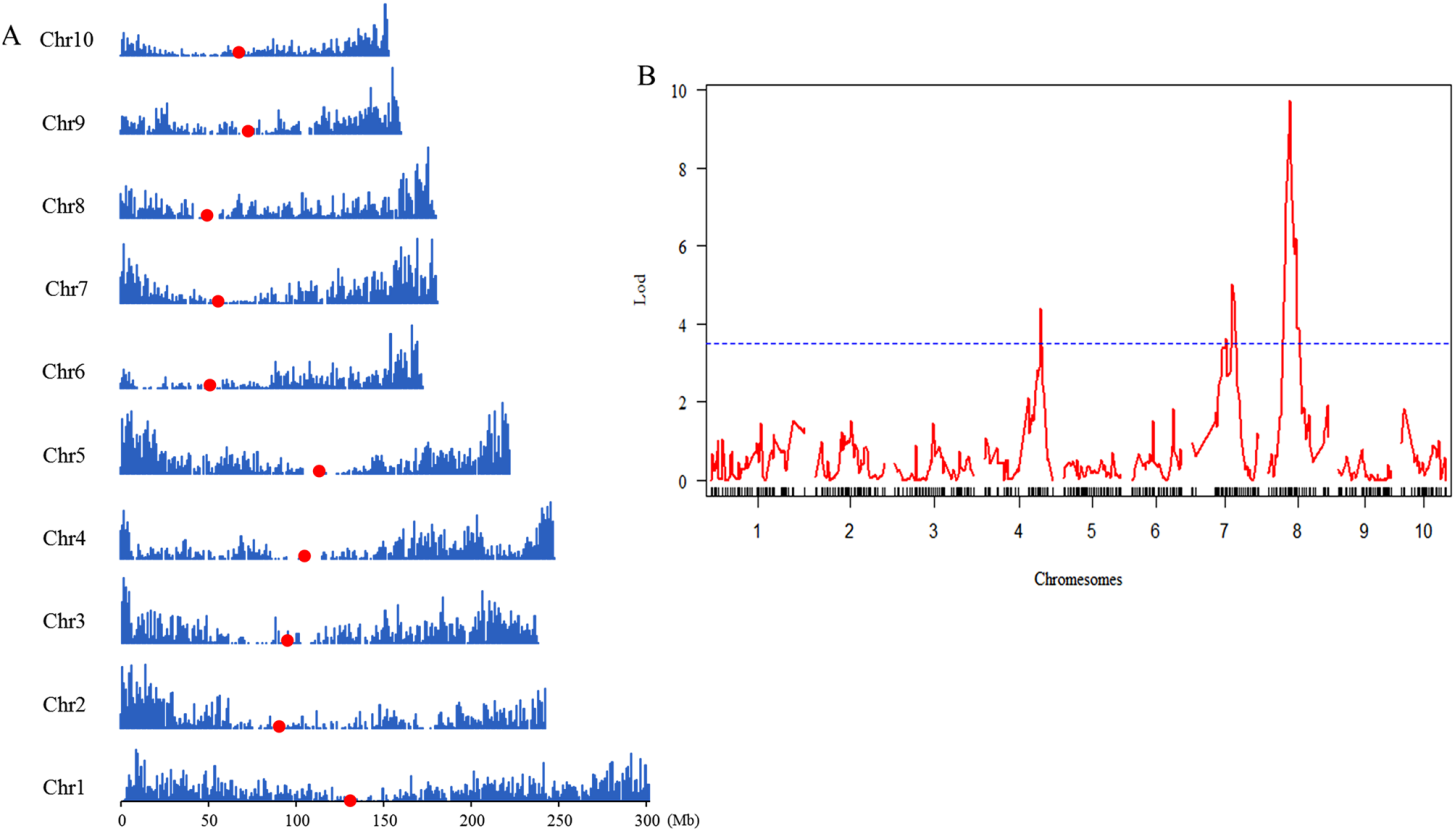
Distribution of 49,608 SNPs identified in Pop6 (**A**) and QTL mapping of TSC resistance in Pop6 (**B**). The red dot represents centromere.

Phenotypic score data for resistance to Tar Spot Complex (TSC) for Pop6, which exhibited with the highest rate of genotyping error among the parental lines, was used to study the impact of genotyping error on linkage analysis. A total of 49,608 unimputed SNP genotypes for each individual were used for linkage analysis, with the highest number of 8242 SNPs on Chr.1 and the lowest number of 3734 SNPs on Chr. 6. These SNPs were not spread out evenly along each chromosome but were distributed at a lower density near the centromeres, indicating that fewer recombination events happened in these regions (Fig. 5A). After bin map construction, 437 bin markers across all 10 maize chromosomes were identified. The number of markers on each chromosome varied from 34 markers on Chr. 10 to 56 markers on Chr. 1. The average genetic distance between markers on each chromosome varied from 2.83 cM on Chr. 5 to 3.94 cM on Chr. 1.

Resistance to TSC were mapped, and three major QTLs for this trait were identified on Chrs. 4, 7 and 8 (Fig. 5B) that together explained 46.11% of the variance in TSC resistance, with the QTL on Chr. 4, 7, and 8, individually explaining 9.36%, 14.12%, and 22.31% of the phenotypic variance for TSC resistance, respectively. The major QTL detected on Chr. 8 exhibited the highest LOD value of 10.17 at 50.00 cM, which was consistent with the result of a previous study^37^, indicating that the influence of the genotyping error due to GBS on QTL mapping could be reduced by using bin markers.

### Genomic prediction using unimputed and imputed SNP data

**Figure 6:**
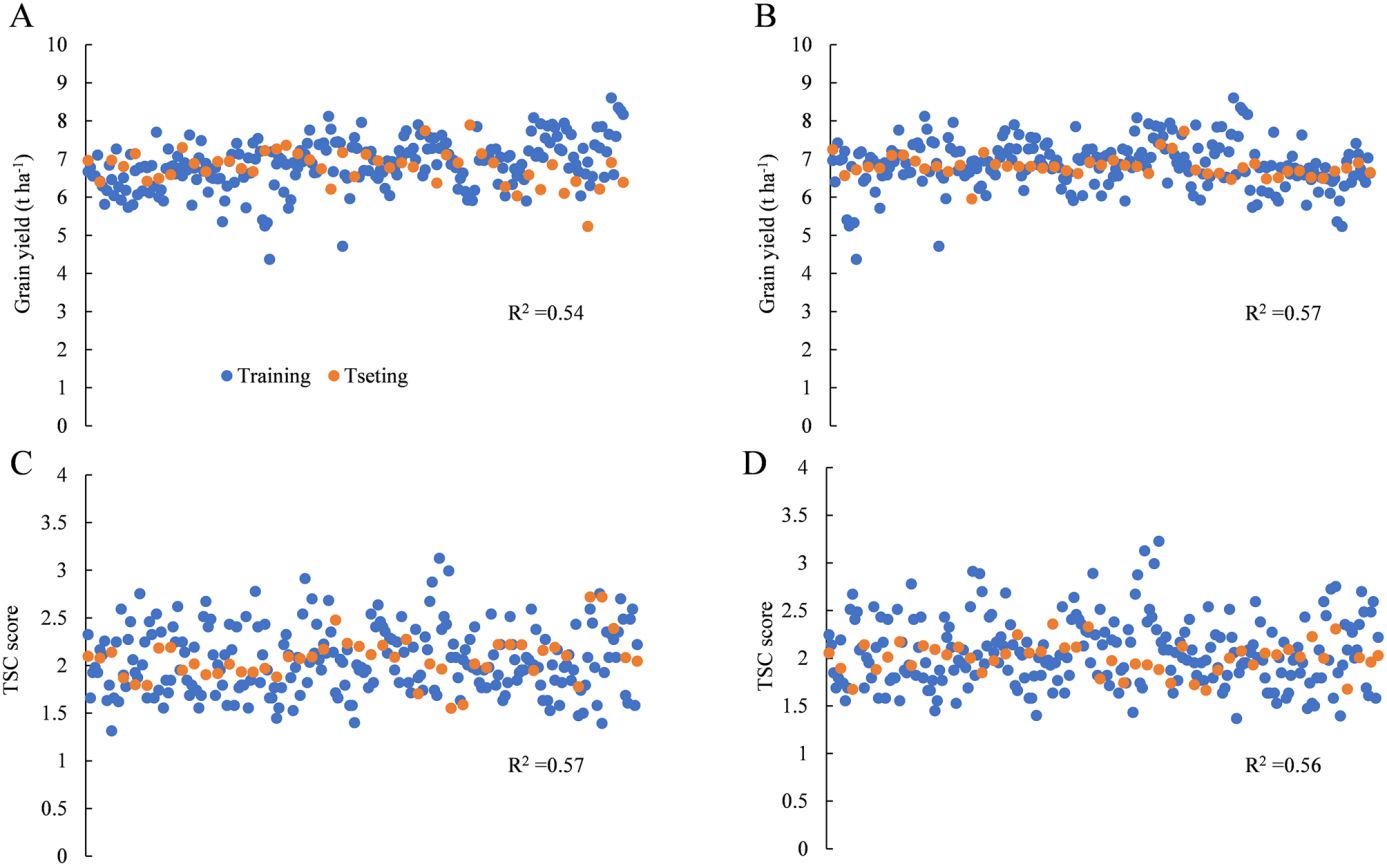
Genomic prediction of GY (**A**, **B**) and TSC resistance (**C**, **D**) using unimputed (**A**, **C**) and imputed (**B**, **D**) SNP data.

In order to understand the effect of imputation on GP accuracy, filtered unimputed (167,617) and imputed (341,312) SNP data for Pop1 were analyzed with phenotypic data for two traits, including GY (H^2^ = 0.84) and TSC (H^2^ = 0.80). The predictions were conducted using the *rrBLUP* package in R software. The average prediction accuracies for GY and TSC resistance was 0.54 ± 0.09, and 0.56 ± 0.08, respectively, when using only unimputed SNP data (Fig. 6A,B). The prediction accuracies for the two traits were about the same (p = 0.16), as the broad-sense heritability of the two traits were similar. When analyzing these traits using the imputed SNP genotype data, the average prediction accuracies for GY and TSC resistance were 0.57 ± 0.09 and 0.56 ± 0.09, respectively (Fig. 6C,D). Prediction accuracy significantly improved when using imputed SNP data to analyze variation in GY (p = 6.17E−03), but no difference in prediction accuracy for TSC resistance (p = 0.63) was found when using imputed SNP data. The difference in prediction accuracies using imputed and unimputed SNP data indicated that for complex traits like GY, more markers would be needed to improve prediction accuracy.

## Discussion

Significant advances in sequencing technology have occurred over the past few years and have led to a better understanding of the maize genome. GBS is a relatively inexpensive method for genotyping large numbers of samples and provides more SNPs than SNP arrays. One of the advantages of GBS is cost-effectiveness; the cost of GBS per marker is less than US$0.001^7^. In our study, one-enzyme based GBS was used to genotype 2240 maize lines from eight tropical maize populations. A total of 995,120 SNPs of raw data was generated for each individual. The genotyping error rate was checked in six bi-parental populations and the average error rates were found to be 0.16% and 0.54% for homozygous and heterozygous loci, respectively (Table 4), which means that ~ 99.9% of the homozygous loci were identical between replications. The error rates for heterozygous loci were about threefold higher than for the homozygous loci. The high error rate for the heterozygous loci together with the error rate of over 8% for the synthetic F1, indicated the need for further improvement in calling of heterozygous loci in GBS. Because the GBS pipeline is designed to favor a higher number of markers at the expense of depth, this system is inherent weaker at calling heterozygotes with high levels of precision. Among inbred samples, both error-prone SNPs and spurious SNPs originating from paralogous tags will appear to be excessively heterozygous^10^. The high rate of missing data is another issue for GBS. In our study, the average rates of missing SNP genotypes were 57.72% and 39.05% for the association panels and the bi-parental populations, respectively (Table 2), mainly due to the inherently low-coverage and multiplex approach for this sequencing method. Therefore, it is essential to develop improved imputation pipelines for GBS data. As shown in this study, two imputation pipelines in TASSEL software were used for genotypic data imputation in both the association panels and bi-parental populations. The number of filtered SNPs doubled for the association panels and increased by an average of 2.50-fold for the bi-parental populations (Tables 2 and 3). Moreover, with the rates of missing SNP genotypes greatly reduced, the error rates were also decreased (Table 4), indicating that the imputation methods we used could efficiently estimate for the missing GBS genotypes.

These GBS SNPs were extensively applied to the genetic characterization of germplasms, population studies, marker-trait association analysis, and marker-assisted breeding. A large number of high-quality SNPs cover the entire genome of maize, which enables a better understanding of the germplasm. In population structure analysis, the subgroups of Pop1 could be separated more clearly than they could by using an oligo pool assay, which offered only 1536 SNPs^33^. On the other hand, few differences were apparent between the unimputed and imputed data for all the populations analyzed by PCA or MDS (Fig. 1–3). Therefore, filtered unimputed data of GBS can be recommended for the population structure in maize. The rate of LD decay is a key factor in GWAS. As an outcrossing species, maize experiences rapid LD decay; therefore, as many as 750,000 markers are required to evaluate all genes simultaneously^38^. LD decay in Pop1 was about 3 kb when estimated using imputed data, which was more rapid than when using unimputed data (Fig. 4A,B). Accordingly, GWAS using imputed data was more powerful for identifying functional genes with reduced noise using more markers (Fig. 4C,D). Moreover, GBS also performed well in GWAS for complex traits, such as grain yield and drought tolerance^38^. For linkage analysis, we used a DH population with low percentage heterozygosity as a case study; and more QTLs for TSC resistance were detected when using GBS than when using low-density SNP markers in the same maize population^39^. GBS was also efficient for linkage analysis in populations with a high percentage of heterozygosity, such as the discovery of genomic regions responsible for resistance to maize lethal necrosis (MLN)^40^. GP, which uses genotypic and phenotypic data to estimate GEBVs, is more effective than conventional phenotypic selection for increasing genetic gain for GY^41^. Prediction accuracy is a key factors for genetic gain calculation. Compared to low-density SNPs, GBS offered better prediction accuracy, especially for traits measured under stressed conditions or those with low or moderate heritability^33^. In our study, prediction accuracies for GY and TSC resistance were 0.54 and 0.57, respectively (Fig. 6), which indicated that our GBS data could efficiently be used to estimating GEBVs for various traits.

GBS enables identification of genome-wide SNPs, but the low sequencing coverage also introduces issues, such as high rates of genotyping error, high rates of missing data, lower accuracy when calling heterozygous SNP calling, and therefore, potentially lower marker density^42^. Several improved GBS approaches have been developed to solve these issues, such as tGBS (tunable GBS), in which only double-digested fragments are amplified and sequenced to increase genotyping accuracy, especially for heterozygous sites, while reducing the missing rate^43^. Managing GBS data using bioinformatic and statistical methods is another way to reduce the quantity of missing data. Many highly accurate methods for data imputation are now available for GBS data^44–47^. In order to increase the number of available SNPs, haplotype information for more than 60,000 temperate and tropical maize germplasms was used as a reference genome instead of the conventional reference genome developed from maize inbred B73. About 150,000 high-quality SNPs were called from the association panels in the present study, sufficient for most types of genetic analyses in maize. Although a large number of SNPs were called, many more markers would be needed for efficient GWAS in maize. Therefore, a more comprehensive maize haplotype map is needed, in addition to bioinformatics pipelines such as PHG^16^.

## Conclusion

Our goal for this study was to summarize various applications of GBS in maize. In the present study, 2240 maize materials from eight populations, including association panels and bi-parental populations with high or low heterozygosity rates, were analyzed using GBS. Approximately one million SNPs that could be used for genetic diversity analysis, GWAS, linkage mapping, and genomic prediction were identified for each individual. Our study provides useful information as to the strengths and constraints of GBS for genetic analyses in maize.

## Materials and methods

### Plant materials

For one association mapping population, we designated tropical maize association panel of 267 inbred lines from the Drought Tolerant Maize for Africa (DTMA) panel as Pop1^34,48^, A second maize association panel of 523 inbred lines of temperate maize from the Ames panel was designated as Pop2. The lines in the Ames panel with clear heterotic group information and off-PVP lines were chosen for the study^35^, including 125 non-stiff stalks, 128 stiff stalks, 72 sweet corns, 53 popcorns, 53 tropical lines and 92 off-PVP lines with no group classification information.

We included three bi-parental populations with high heterozygosity rates, including Pop3, a BC1F2 population of 174 families created by crossing a drought-tolerant donor line DTPC9F104 with an elite inbred line CML491. The BC1 population, Pop4, was generated by crossing CML444 and CKL09001; this population was developed through the Water Efficient Maize for Africa (WEMA) Project. The F2 population, Pop5, containing 423 individuals derived from the cross between CML494 and CLWN201, was developed under the Improved Maize for African Soils (IMAS) Project.

A set of three bi-parental populations with low heterozygosity rates were also used in the present study. The DH population, Pop6, of 209 individuals, which has been described in a previous study^37^, was generated by crossing LPSC7F64 and CML495. A RIL population, Pop7, was a nested association mapping (NAM) population from a cross between the parents B73 and CML247. Finally, Pop8, included 285 lines of the intermated B73 × Mo17 (IBM) population.

### Genotyping-by-sequencing, SNP calling, and data imputation

SNP calling and imputation were conducted at Cornell University. The leaves of all materials were sampled at the seedling stage. The DNA extraction was performed using one leaf per plant with the CTAB method. The DNA isolated from each sample was digested by the type II restriction endonuclease *Ape* KI and 4 to 8 bp barcode adapters were added separately to the 3′ ends of the top strands and 5′ ends of the bottom strands. DNA libraries for each sample were then constructed and sequenced following the GBS protocol^9^. Sequences from Pop4, Pop7, and Pop8 were collected in lanes of a single flow cell at 348-plex, while sequences for other populations were collected at 96-plex. For each lane, about 2090 Mbp of data were obtained with the default read length of 64 bp^9^.

Raw data in a FASTQ file was then used for SNP calling, together with the barcode information and Tags On Physical Map (TOPM) data, which contained SNP position information. We used TOPM data from AllZeaGBSv2.7 downloaded from Panzea (https://www.panzea.org/), which contained information for 955,690 SNPs mapped with B73 AGPv2 coordinates. SNP calling was then performed using the TASSEL-GBS pipeline^10^. Reads were first filtered according to the barcode matches and missing numbers with the minimum Kmer count set to 10. The same reads then clustered together as a unique tag. Loci with a tag alignment value higher than the gap alignment threshold (ratio of InDel contrasts to non-InDel contrasts) of 1.0 were then excluded from the pool. SNPs were called with the average sequencing error rate per base set to 0.01, and the minimum quality score for a SNP position set to zero. Genotype data for each sample was then obtained from a set 955,690 SNPs, among which the position information for 570 SNPs was unclear. Finally, 955,120 SNPs from all ten chromosomes for each individual were used for further analysis.

After SNP calling, missing data were imputed using TASSEL 5.0 software^49^. Either FSFHap for full-sib families or FILLIN for inbred lines were used for imputation of GBS data^15^. FILLIN was based on haplotype information. Haplotype block sizes at sites were set to 8000, the minimum number of informative minor alleles in the search window was set to 20, and the maximum error rates for applying one haplotype or the Viterbi algorithm with two haplotypes to an entire site window were set to 0.01 and 0.003, separately.

### Genotyping error

For the bi-parental populations, the parental lines together with an equally mixed sample of parental lines (considered as F1 only for Pop3 and Pop5) were sequenced two or more times at different levels of multiplexing to calculate the error rate of sequencing. The missing data were removed first. Then the proportions of loci between replications were designated as an error rate. The error rate for the entire data and for only homozygous loci were calculated separately.

### SNP filtering in each population

Unimputed data for each population were filtered as follows: (1) For the association panels, first the inbred lines with SNP heterozygosity rate over 5% and missing genotype rate over 40% for the 96-plex data or 75% for the 384-plex data were removed. Then, the SNPs were filtered with MAF > 0.05 and SNP calling rate > 50% were filtered out. (2) For the bi-parental populations, first the sites that were polymorphic in the parents without missing genotypes were identified. Then the individuals from Pop6, Pop7, and Pop8 were filtered in the same manner as the inbred lines in the association panels. However, individuals of Pop3, Pop4, and Pop5 were only removed if their missing genotype rate exceed 40% for the 96-plex data or 75% for the-384 plex data, as all of these individuals exhibited high rates of heterozygosity. Finally, the SNP of the six bi-parental populations were filtered in the same manner as the association panels by eliminating any SNPs with MAF > 0.05 and SNP calling rate > 50%.

After imputation, data for all populations were again filtered. After filtering, the imputed data for samples from each population were consistent with the unimputed data. SNPs with MAF > 0.05 and SNP calling rate > 50% were filtered out, just as for the unimputed data.

### Population structure analysis

Principal component analysis (PCA) was performed in the two association panels using TASSEL software with a covariance matrix of both filtered unimputed and imputed SNP data sets. Five major components were identified for each population. The first two major components with highest variants explanation levels were used.

Principal coordinate analysis or multidimensional scaling (MDS) of six bi-parental populations were performed in TASSEL using both filtered unimputed and imputed SNP databases. The MDS analysis started with a distance matrix calculated using identity-by-state similarity; the results were similar to PCA.

### LD and association mapping analyses

In the tropical maize association mapping panel, 167,617 unimputed and 341,312 imputed SNPs were used separately for LD and association mapping analysis, respectively. LD between SNPs was first calculated in TASSEL 5.0 software using the Sliding Window method with the LD window size set to 50. LD across all chromosomes was then calculated using R software.

Data for kernel color in Pop1 was used for GWAS. Among the 242 inbred lines, 159 lines had yellow kernels and the remaining lines had white kernels. A GLM (PCA) was used to identify SNPs associated with kernel color in TASSEL 5.0 software. A Bonferroni correction (0.01/n, where n equals number of SNPs) was used to detect significant association signals with thresholds of – log10(P) > 7.22 and – log10(P) > 7.53 for the unimputed and imputed data, separately.

### Linkage mapping analysis

TSC disease resistance score data in the DH population Pop6 from Cao et al.^37^ were used to perform linkage analysis. We used a set of 49,608 unimputed SNPs that were was further filtered as follows: (i) the similarity rate of SNPs within a window size of eight was calculated and the unlinked SNPs were removed (similar rate < 95%); (ii) then each bin was merged with any linked high-quality consecutive SNPs; (iii) finally, each bin was treated as a marker for constructing a genetic map.

The length of the resulting genetic map was 987.35 cM with 437 bin markers and an average marker density was of 2.26 cM. QTLs for TSC resistance were detected using the composite composition-interval mapping method in *R/qtl* package with threshold LOD scores of 3.5.

### Genomic prediction

The phenotypic data for grain yield (GY) and TSC disease scores of the Pop1 panel were collected from Cairns et al.^48^ and Cao et al.^37^, separately. The GY of Pop1 were collected in Mexico in 2008, 2009, and 2010 and Thailand in 2009 and 2010. Average GY ranged from 4.37 to 8.60 t ha^−1^ with an overall average of 6.84 t ha^−1^.

GP was performed using the *rrBLUP* package^50^ in Pop1. A five-fold cross validation was used to estimate prediction accuracy with 100 replications. The correlation between the predicted yield values and the observed yield values represented prediction accuracy. In order to study the effect of SNP imputation on prediction accuracy, 167,617 unimputed and 341,312 imputed SNPs were used for GP.

## Data Availability

The datasets used and/or analyzed during the current study are available from the corresponding author upon reasonable request.
